# Aspartate Transaminase AST2 Involved in Sporulation and Necrotrophic Pathogenesis in the Hemibiotrophs *Magnaporthe oryzae* and *Colletotrichum graminicola*

**DOI:** 10.3389/fmicb.2022.864866

**Published:** 2022-04-11

**Authors:** Penghui Zhang, Zhenyu Fang, Yanyue Song, Shaowei Wang, Lina Bao, Mingyu Liu, Yuejia Dang, Yi Wei, Shi-Hong Zhang

**Affiliations:** ^1^College of Plant Sciences, Jilin University, Changchun, China; ^2^College of Plant Protection, Shenyang Agricultural University, Shenyang, China

**Keywords:** aspartate transaminase (AST2), conidiogenesis, necrotrophic pathogenesis, *Magnaporthe oryzae*, *Colletotrichum graminicola*

## Abstract

Aspartate family includes five additional amino acids other than aspartate, among which most except aspartate have been reported for their action in pathogenesis by amino acid biosynthesis. However, how aspartate, the initial substrate of this family metabolic pathway, is involved in pathogenesis remains unknown. Here, we focused on aspartate transaminase (AST) that catalyzes transamination reaction between glutamate-aspartate in *Magnaporthe oryzae*. Three *MoAST* genes were bioinformatically analyzed, of which *MoAST2* was uniquely upregulated when invasive hyphae switched to necrotrophic pathogenesis. *MoAST2* deletion (Δ*Moast2*) caused a drastic reduction in conidiogenesis and appressorium formation. Particularly, Δ*Moast2* was observed to be proliferated at the biotrophic phase but inhibited at the necrotrophic stage, and with invisible symptoms detected, suggesting a critical role in necrotrophic phase. Glutamate family restored the Δ*Moast2* defects but aspartate family did not, inferring that transamination occurs from aspartate to glutamine. MoAST2 is cytosolic and possessed H_2_O_2_ stress tolerance. In parallel, *Colletotrichum graminicola* AST2, CgAST2 was proven to be a player in necrotrophic anthracnose development. Therefore, conserved AST2 is qualified to be a drug target for disease control.

## Introduction

The hemibiotrophic filamentous fungus, *Magnaporthe oryzae*, is a devastating pathogen that attacks most crops, involving rice and wheat, thus posing a major challenge to global food security. The rice blast disease cycled in a way of the sequential process, which involves attachment of a conidium to the leaf surface, conidial tube germination, appressorium differentiation and maturation, penetration peg formation, invasive hyphal proliferation, necrotic lesion development, conidiophore stalk differentiation, and sporulation (Wilson and Talbot, 2009). To a typical hemibiotrophic pathogen, the whole pathogenesis can be divided into biotrophic and necrotrophic stages. Rice blast fungus initially adopts a biotrophic infection strategy, which lasts approximately 3–5 days, and the fungus colonizes the living host cells without causing visible damage to the host at this stage. Later it enters into a devastating necrotrophic phase, where the fungus rapidly destroys the infected host tissue (Kankanala et al., 2007).

Like the rice blast fungus, *Colletotrichum graminicola* is also a hemibiotrophic filamentous fungus that leads to the maize anthracnose disease and poses a threat to global food security (Deising et al., 2000; Münch et al., 2008, 2011; Dean et al., 2012; Ludwig et al., 2014). To establish disease, *C. graminicola* sequentially differentiates highly specific infection structures. Attached on the cuticle of maize leaves, lunate conidia germinate to form dome-shaped melanized appressoria and penetration pegs, which generate enormous turgor pressure and access to the epidermal host cells (Bechinger et al., 1999; Deising et al., 2000). Subsequently, voluminous infection vesicles and primary hyphae biotrophic infection structures were differentiated in the epidermal host cells. At this biotrophic stage, macroscopically visible symptoms do not happen, but after fungus switches to form highly destructive secondary hyphae, which rapidly colonize and kill the host tissue (Horbach et al., 2011). Due to the economic significance and genetic tractability, both the pathogenic fungi *M. oryzae* and *C. graminicola* have been intensively studied as model organisms (Deising et al., 2000; Talbot, 2003).

As important decomposers, most fungi, including *M. oryzae* or *C. graminicola*, possess strong metabolic and synthetic abilities. They are capable of synthesizing all amino acids, purines, and pyrimidines *de novo* and then growing axenically in synthetic minimal media (MM), which contains minimal carbon and nitrogen sources (Pennisi, 2010). However, in the plant–pathogen interaction system, fungal pathogens frequently encounter nitrogen starvation because some plant amino acids are not available for fungal nutrition and some nitrogen resources such as cysteine, methionine, tryptophan, histidine, and arginine are present in trace amounts in the leaf apoplast (Solomon et al., 2003; Donofrio et al., 2006; Fernandez et al., 2014). Glutamate or aspartate, as a basic biological nitrogen donor, is abundant in apoplast space, based on which fungal invasive hyphae are expected to synthesize those important but deficient amino acids for growth and development.

**FIGURE 1:**
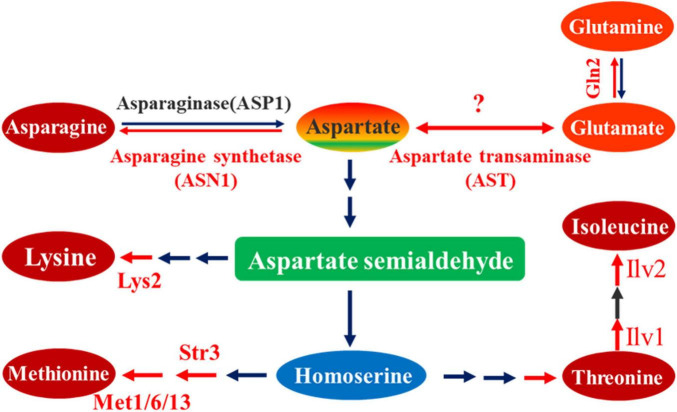
Metabolic pathway of aspartate as substrate. In this metabolic pathway, aspartate is the initial substrate, from which different biosynthesis reaction steps occur, yielding terminal products including asparagine, lysine, methionine, threonine, and isoleucine. According to the reversible reaction between aspartate and glutamate, the aspartate transaminase is also indicated in this pathway. The red arrow line represents a biosynthesis reaction marked with the corresponding catalytic enzyme, which has been proved to be associated with pathogenesis; black arrow line represents a biosynthesis reaction not studied or unrelated to pathogenesis.

However, to resist host plant innate immunity and maintain energy homeostasis for invasive hyphae growth, pathogenic development, and sporulation, fungal pathogens must initiate all available active processes to require greater nutrient acquisition from the host plant (Wilson et al., 2012; Saint-Macary et al., 2015). Amino acid metabolic pathways have been demonstrated to play an essential role in the process of growth, conidiogenesis, and pathogenesis in pathogenic fungi. Cystathionine β-lyase (Str3), cystathionine γ-synthase (met1), methionine synthase (met6), and methylenetetrahydrofolate reductase (met13) are important enzymes responsible for the catalytic function of different steps in methionine biosynthesis. Deletion of one of these enzyme genes led to a drastic reduction in pathogenicity of *M. oryzae* (Wilson et al., 2012; Yan et al., 2013; Saint-Macary et al., 2015). Amino acid reductase Lys2 was involved in the lysine biosynthesis of *Penicillium chrysogenum* but also promoted the production of the secondary metabolite penicillin. In *M. oryzae*, Lys2 was necessary for lysine biosynthesis that affected growth, conidiogenesis, and pathogenicity of the fungus (Chen et al., 2014). Acetolactate synthase catalyzes the first common step in isoleucine biosynthesis. The catalytic subunit Ilv2 and regulatory subunit Ilv6 playing essential roles in isoleucine biosynthesis are important for conidial morphogenesis, appressorial penetration, and pathogenicity (Du et al., 2013). Similarly, the threonine dehydratase, MoIlv1, involved in isoleucine biosynthesis, is also relevant to morphogenesis, appressorium formation, invasive hyphae growth, and pathogenicity (Du et al., 2014). The asparagine synthetase Asn1 is required for asparagine production from aspartate and glutamine, the sole pathway to *de novo* asparagine biosynthesis in *M. oryzae*. Asn1 deletion mutant strains could not grow on minimal media without asparagine supplementation, the biotrophic growth was aborted, and the asn1 deletion strains were non-pathogenic (Marroquin-Guzman et al., 2018). Biosynthetically, all the studied metabolic processes involve biosynthesis of lysine, methionine, threonine, isoleucine, and asparagine, which are all derived from aspartate and belong to the terminal products of aspartate family (Figure 1). Therefore, aspartate metabolic pathway is required for pathogenesis in *M. oryzae*. However, as an initial nitrogen donor of other amino acid biosynthesis, aspartate and its biosynthesis remain elusive in *M. oryzae* and other plant fungal pathogens.

Most amino acids are synthesized from α-ketoacids and later transaminated from another amino acid, usually glutamate. The enzyme involved in this reaction is an aminotransferase. Glutamate dehydrogenase catalyzes the reductive amination of α-ketoglutarate to glutamate. A transamination reaction takes place in the synthesis of most amino acids. Aspartate family of amino acids involves threonine, lysine, methionine, isoleucine, asparagine, as well as aspartate. The biosynthesis of aspartate is a one-step reversible reaction that is catalyzed by a single enzyme aspartate transaminase (AST) (Figure 1). Aspartate transaminase catalyzes the transfer of an amino group from glutamate onto α-ketoglutarate to yield aspartate and oxaloacetate. In physiological conditions, however, the reaction also runs in the opposite direction from aspartate onto α-ketoglutarate to yield glutamate and oxaloacetate. Aspartate and glutamate, together with oxaloacetate and α-ketoglutarate, link amino acid metabolism to Citric Acid Cycle through catalysis of aspartate transaminase. Recently, cytoplastic MoGln2, the *M. oryzae* glutamine synthetase that catalyzes the synthesis of glutamine, was shown to be important for vegetative growth, conidiation, appressorium formation, maintenance of cell wall integrity, oxidative stress tolerance, and pathogenesis in *M. oryzae* (Aron et al., 2021a), further implying the role of aspartate in pathogenesis of *M. oryzae*. In this study, we focused on the *AST2*, the upregulated yeast *AST2* homolog in rice blast fungus. Through constructing gene deletion mutant strains, we established a series of biological research systems for *AST2* analysis. Our research reveals that the *MoAST2* is important for aspartate metabolism and glutamate yield, exerting a significant effect on conidiogenesis and necrotrophic pathogenesis in *M. oryzae*; and that *CgAST2* played a similar role in necrotrophic anthracnose development in *C. graminicola.* Our findings, therefore, suggest that glutamine biosynthesis mediated by *AST2* provides a key target for antifungal design against plant fungal pathogens.

## Results

### Genetic Relationship and Expression Patterns of *MoAST1, −2*, and *−3* and *CgAST2*

**FIGURE 2:**
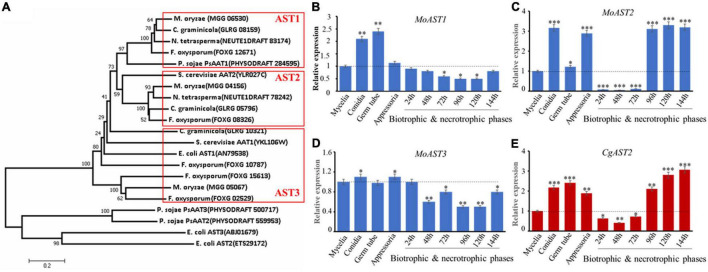
Phylogenetic analysis and expression patterns of ASTs. **(A)** Phylogenetic tree of the three ASTs of *M. oryzae* and the corresponding homologs in *C. graminicola* and other organisms. The phylogenetic tree was constructed using the neighbor-joining algorithm in MEGA7.0.9 with similar sequences from the aspartate transaminase family. The full protein sequences from different organisms analyzed were as follows: these proteins (the accession numbers) include *M. oryzae* (MoAST1, MGG_06530; MoAST2, MGG_04156; MoAST3, MGG_05067); *S. cerevisiae* (AAT1, YKL106W; AAT2, YLR027C); *C. graminicola* (AST1, GLRG_08159; AST2, GLRG_05796; AST3, GLRG_10321); *F. oxysporum* (ATS1, FOXG_12671; ATS2, FOXG_08326; ATS3, FOXG_02529); *P. sojae* (PsAAT1, PHYSODRAFT_284595; PsAAT2, PHYSODRAFT_559953; PsAAT3, PHYSODRAFT_500717); *N. tetrasperma* (AST1, NEUTE1DRAFT_83174; AST2, NEUTE1DRAFT_78242); *E. coli* (AST1, AN79538; ATS2, ETS29172; ATS3, ABJ01679); *A. thaliana* (AAT1, AT2G30970; AAT2, AT5G19550; AAT3, AT5G11520); and *O. sativa* (AAT1, BAA23815; AAT2, ABL74572). Expression patterns of *MoAST1*, *MoAST2*, *MoAST3*, and *CgAST2* were performed during growth, development, and pathogenesis. **(B)** Expression profile of *MoAST1.* **(C)** Expression profile of *MoAST2.* **(D)** Expression profile of *MoAST3.* **(E)** Expression profile of *CgAST2.* The expression of the four *AST* genes was quantified by quantitative real-time qPCR after synthesis of cDNA in each developmental stage. The *M. oryzae ACTIN* gene (MGG_03982) or *C. graminicola* ACTIN gene (GLRG_03056) was used for internal control for normalization, and the expression level of each gene at the mycelial stage was defined to be one for further comparisons. The qPCR results were obtained from three independent biological replications with three technical replicates. Error bars represent standard deviations. Asterisks indicate statistically significant differences (*p < 0.05; **p < 0.01; ***p < 0.001. Data represent the means ± standard deviation from three independent experiments in which triplicate plates were examined for each strain in each experiment).

In the Ensembl Fungi release 52 (Dec 2021 ©EMBL-EBI) resource section for *M. oryzae*,^1^ the amino acid sequences of both aspartate transaminase AAT1(YKL106W) and AAT2 (YLR027C) from the brewer’s yeast *Saccharamycess cerevisiae* were used to search the aspartate transaminases (ASTs) of *M. oryzae*. Three putative amino acid sequences that encode ASTs were found, termed as MoAST1 (MGG_06530), MoAST2 (MGG_04156), and MoAST3 (MGG_05067), respectively. The three obtained MoAST amino acids were used for a blastP search to identify other interested AST amino acid sequences in National Centre of Biotechnology Information.^2^ The established phylogenetic tree showed two major clades among the selected proteins. All the fungal and yeast ASTs are in one clade, and AST1 proteins and AST2 proteins are in two neighbor-jointing subclades, respectively. MoAST3 together with the so-called CgAST3 is significantly far from AST1 and AST2 in genetic relationship (Figure 2A). Further analysis revealed that MoAST1, MoAST2, and MoAST3 shared a close ancestor with CgAST1, CgAST2, and CgAST3 of *C. graminicola* (Cg), respectively (Figure 2A). In addition, MoAST2 shares 78% identity and 88% similarity with CgAST2, and shares 71% identity and 82% similarity with FoAST2, and 52% identity and 66% similarity with ScAST2, suggesting a comparatively close genetic relationship with filamentous fungal phytopathogens.

To evaluate the biological activities of the three *MoAST* genes, we analyzed the abundance of *MoAST1, MoAST2*, and *MoAST3* transcripts at different stages of *M. oryzae* development and pathogenesis. In comparison with that of the mycelia, *MoAST1* expression was relatively higher at conidial, and appressorial stages but was downregulated and at low levels during *in planta* infection from 24 h post-inoculation (hpi) to 144 hpi (Figure 2B). *MoAST3* expression remained almost unchanged at cultivate stage prior to infection except for a significant increase at conidial and appressorial stages but downregulated and was in extremely significant low levels at earlier infection stage (Figure 2D). Compared with *MoAST1* and *MoAST3*, *MoAST2* was drastically upregulated during appressorial formation and maturation and showed up to a three-fold increase as the appressoria matured (Figure 2C); from 24 to 72 hpi, *MoAST2* was sharply downregulated to an almost undetected expression level; however, from 96 to 144 hpi, it returned to higher level in the appressoria (Figure 2C). Interestingly, *C. graminicola AST2* possessed a similar expression profile with *MoAST2* (Figure 2E). Therefore, *MoAST2* together with the *CgAST2* was reckoned to be a pathogenic related factor. Thereafter, we bioinformatically identified the *MoAST2.*

**FIGURE 3:**
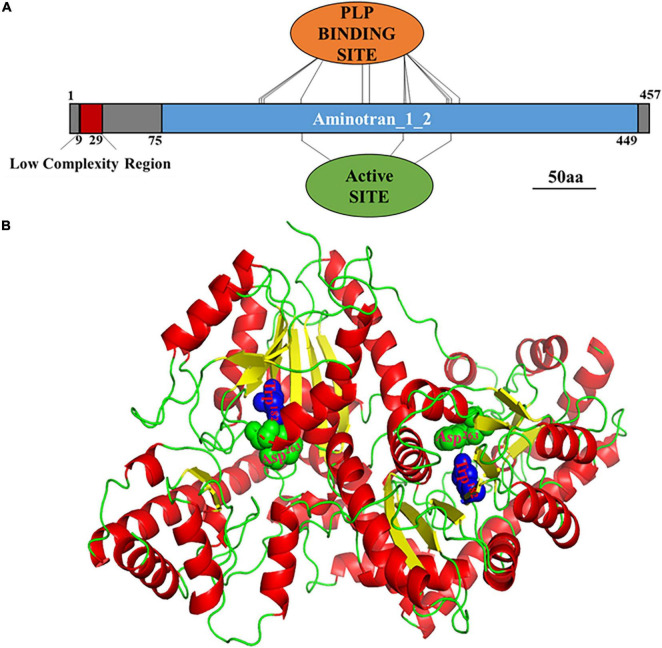
Prediction of *MoAST2* structure. **(A)** Schematic representation of domains identified in MoAST2. From 9 to 29nt, Low complexity region: LCRs are very important in phenotypic plasticity, which play an active role of positive and purifying selection in their evolution. Thirteen pyridoxal 5′-phosphate (PLP) binding sites, including 150, 151, 152, 182, 228, 229, 235, 259, 264, 266, 298, 299, and 307, were found. Pyridoxal phosphate is the active form of vitamin B6 (pyridoxine or pyridoxal). PLP is a versatile catalyst, acting as a coenzyme in a multitude of reactions, including the biosynthesis of amino acids and amino acid-derived metabolites. These sites can bind with PLP. Active sites (Trp^182^, Asp^263^) and PLP enzymes exist in their resting state as a Schiff base, the aldehyde group of PLP forming a linkage with the epsilon-amino group of an active site lysine residue on the enzyme. The alpha-amino group of the substrate displaces the lysine epsilon-amino group, in the process forming a new aldimine with the substrate. This aldimine is the common central intermediate for all PLP-catalyzed reactions, enzymatic and non-enzymatic. 75-449, Aminotransferase class I/classII: Aspartate aminotransferase (AAT) superfamily (fold type I) of pyridoxal phosphate (PLP)-dependent enzymes. **(B)** Three-dimensional (3-D) structures of the full length of MoAST2. A homodimer consisting of the two full-length MoAST2 as monomers was viewed in an appropriate direction. Four active sites in the vicinity of subunit interfaces were marked in green and blue.

Based on the genomic DNA sequence, the *MoAST2* gene is located on the *Magnaporthe* chromosome 6, with two introns and a length of 1374 bp open reading frame (ORF) encoding a protein of 457 amino acids. MoAST2 contains one predicted aminotransferase catalytic domain (EC Number: 2.6.1.1), together with which 13 of PLP binding sites and 2 of catalytic active sites were predicted (Figure 3A).

The tertiary (3D) structure of MoAST2 protein was predicted based on the web-based server I-TASSER.^3^ Among the studied ASTs, although there are variations in sequence identities, the overall domain folds of ASTs are highly similar. Like the SpAST protein structure (Jeong et al., 2019), the MoAST2 protein forms a very stable homodimer with an unusually extended interface (Figure 3B). The subunits of the homodimer are tightly combined in the exactly opposite direction through the salt bridges and hydrogen bonds present between side chains as well as between the main chain and the side chain, and many hydrophobic residues are aligned at the interface contributing hydrophobic interaction between two molecules of dimer. The MoAST2 homodimer contains four active sites in the vicinity of subunit interfaces (Figure 3B), which are associated with binding to the cofactor PLP and substrate. According to this structure with active and binding site residues, it appears that aspartate and α-ketoglutarate as substrates bound to the MoAST2 at the binding sites cause the catalytic amino acid transaminase reaction.

### MoAST2 Is Cytosolic and Required for Conidiation

Aspartate transaminases (ASTs) reported were subcellularly localized in cytoplasm or mitochondria (Jaussi et al., 1982). When MoAST2 protein sequence was input in the Euk-mPLoc 2.0 web system^4^ performing the prediction of subcellular localization, MoAST2 was specifically localized in cytoplasm. Further confirmation was carried out through fluorescent microscopic observation of the DsRED or GFP tagged MoAST2 proteins.

**FIGURE 4:**
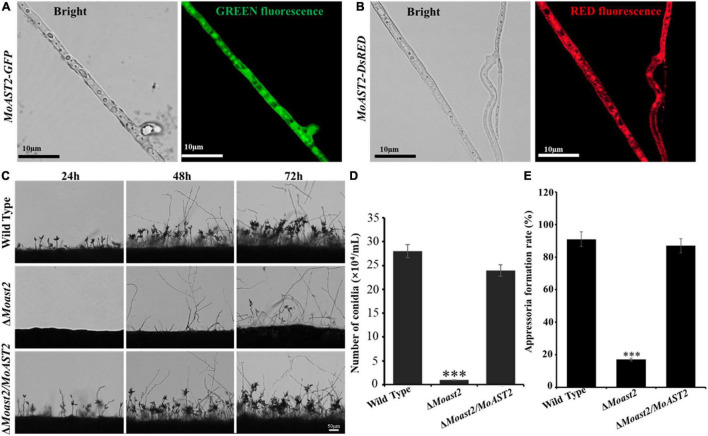
Cytosolic MoAST2 involved in conidial production and appressorium development. **(A)** Localization of MoAST2-GFP and **(B)** DsRED-MoAST2 were examined by Nikon laser confocal. MoAST2-GFP proteins were detected with rather strong green fluorescence signal in cytoplasm of growing hyphae, which was confirmed by DsRED-MoAST2, scale bar = 10 μm. Conidia of the wild-type and the two created strains (Δ*MoAST2*, Δ*Moast2/*Δ*MoAST2*) from 10-day-old tomato oatmeal agar (OTA) were transferred to cover slips, induced for 24, 48, or 48 h. Development of *MoAST2* conidia on conidiophores **(C)** was observed under light microscope. **(D)** Statistical analysis of conidial productivity. The conidia were harvested from the 10-day-old colonies grown on OTA media, and counted using a hemocytometer for all the five strains. **(E)** Appressorial formation rate. Appressorial formation was measured on hydrophobic cover slips and Gel-bond films and was calculated under the microscope at 10 h per inoculation. Bar = 50 μm. Asterisks indicate statistically significant differences (***p < 0.001; data represent the means ± standard deviation from three independent experiments in which triplicate plates were examined for each strain in each experiment).

We constructed a gene deletion vector for targeted gene replacement of the *MoAST2* gene (Supplementary Figure 1A). Then, the deletion mutant strains (Δ*Moast2*) were created by replacing the *MoAST2* ORF with the hygromycin phosphotransferase (*HPH*) gene. Based on the Δ*Moast2* strain, we subsequently created the complementary stain with a *MoAST2-GFP* fusion gene driven by its native promoter (Supplementary Figure 1B). Fluorescent microscopic observation was carried out in the growing hyphae (6d). Green fluorescence signals of the MoAST2-GFP protein were detected with a rather strong green fluorescence signal in growing hyphae (Figure 4A). By comparison, in the wild-type or untransformed strains, the background green fluorescence was too weak to be detected (data not shown). In parallel, we generated the complementary stain of Δ*Moast2* harboring DsRED-tagged MoAST2 in the Δ*Moast2* background (Δ*Moast2*/*DsRED-MoAST2*) (Supplementary Figure 1C). The DsRED fluorescence in the hyphae of Δ*Moast2*/*DsRED-MoAST2* indicated that the DsRED-tagged MoAST2 proteins were distributed in the cytoplasm (Figure 4B), confirming the cytosolic protein of MoAST2.

To investigate the contribution of *MoAST2* gene to vegetative growth of *M. oryzae*, we inoculated the Δ*Moast2*, Δ*Moast2*/*MoAST2*, and the wild-type strains on complete medium (CM). After 7 days of cultivation, mycelial growth was analyzed according to the colony diameter among the three strains. As a result, the growth of Δ*Moast2* strains was reduced to a certain extent in CM (Supplementary Figures 2A,B); but no colony morphology alterations were observed except that the Δ*Moast2* strains appeared to be lower pigmentation on CM solid and liquid media than the wild-type and the complemented strains (Supplementary Figures 2C,D).

To analyze the role of the *MoAST2* gene in sporulation in the rice blast fungus, the Δ*Moast2*, Δ*Moast2/MoAST2*, and wild-type strains were cultured on OMA plates for 10 days, and then conidiophore stalk and conidia were observed on microscope. The result indicated that the Δ*Moast2* strains hardly produced conidia on OMA plates; however, Δ*Moast2/MoAST2* produced more conidia like the wild type (Figures 4C,D). To harvest enough conidia for inoculation assays, we had to prepare for 20 times of wild type plates. Although Δ*Moast2* strains were impaired in sporulation, their germination was normal. In terms of appressorium formation, Δ*Moast2* was severely affected despite being induced by artificial hydrophobic film or by onion epidermis surface (Figure 4E). These data suggest that *MoAST2* is involved in fungal growth and development prior to plant infection.

### Δ*Moast2* Becomes Incompetent When It Switches to Necrotrophic Stage

**FIGURE 5:**
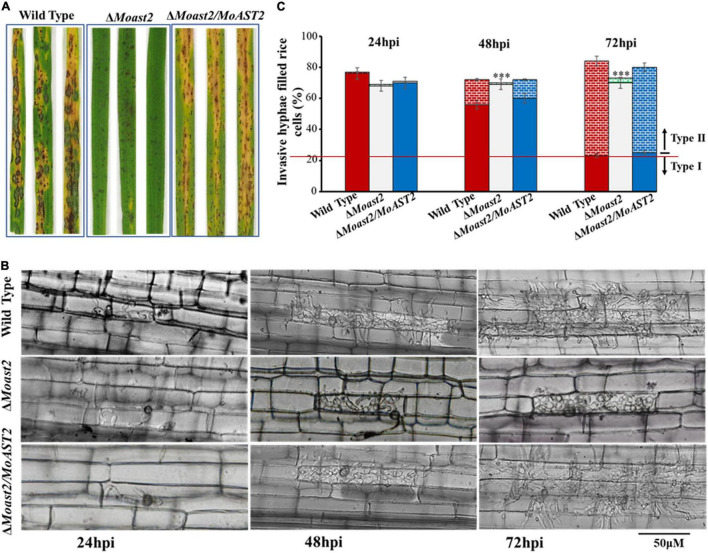
The loss of the *MoAST2* gene has a negative effect on infectious growth. **(A)** Disease symptoms after the inoculation of leaves by spraying with conidia (1 × 10^5^/ml). Diseased leaves were photographed on 7–9 days post-inoculation. **(B)** Rice leaf sheath infection assay. The conidial suspension of indicated strains was dropped onto a rice sheath. Representative photographs of infectious hyphae growing in the cell invaded first (type I) or extending into neighboring cells (type II) were taken after 24, 48, and 72 h of incubation at 25°C. A bar indicates 50 μm. **(C)** The infection rate was calculated according to the number of type I and type II events. The infection status of more than 100 appressoria per leaf sheath was scored at 24, 48, and 72 h post-inoculation. Values represent the averages of five measurements ± standard deviation. Asterisks indicate statistically significant differences (***p < 0.001; Data represent the means ± standard deviation from three independent experiments in which triplicate plates were examined for each strain in each experiment).

To understand the effects of the *MoAST2* on infection, we inoculated intact rice leaves with spores of the wild type, Δ*Moast2/MoAST2*, and *MoAST2*. Δ*Moast2* caused restricted lesions on the compatible cultivar at 144 hpi, although the wild type and Δ*Moast2/MoAST2* have resulted in enlarged lesions (Figure 5A).

To observe the infection process during plant–pathogen interaction, we performed rice leaf sheath assays. At 24 hpi, the wild type and Δ*Moast2/MoAST2* had invaded more than 70% of rice cells; similarly, nearly 70% of rice cells had been invaded (Figures 5B,C). By 48 h, the three strains, Δ*Moast2*,Δ*Moast2/MoAST2*, and wild type were all capable of elaborating invasive hyphae in the first infected cell. By 72 h, the majority of wild type and Δ*Moast2/MoAST2* have spread to the adjacent cells around the infection sites, but the invasive hyphae of Δ*Moast2* were still restricted to the primary infected rice cells (Figures 5B,C). These data indicated that the Δ*Moast2* was unaffected during earlier biotrophic development but switched to be defective at the necrotrophic phase, which may be directly responsible for the reduced lesion size or limited necrotic zones. Therefore, MoAST2 as a player in host–pathogen interactions contributes to the development of rice blast disease.

### Sensitivity of MoAST2 to H_2_O_2_ but Not to Cell Wall Integrity Stressors

**FIGURE 6:**
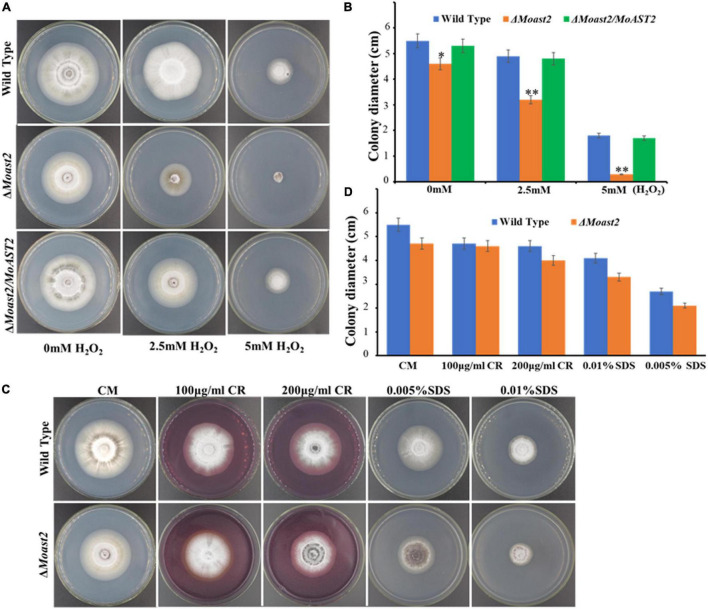
Mycelium growth assay under different stressors. **(A)** H_2_O_2_ sensitivity. The tested strains were cultured on CM supplemented with or without a 2.5 or 5 mM H_2_O_2_ for 7 days. **(B)** The colony diameters of the strain tested were measured, and statistical analysis was performed. **(C)** The wild-type and *MoAST2* mutants were cultured on CM medium supplemented with (200 μg/ml CR and 0.01% SDS) at 28°C for 8 days before being photographed. **(D)** Inhibition rate of WT and mutant strains. Statistical results for growth inhibition rate were obtained from at least three independent replicates. Error bars represent standard deviations. Asterisks indicate statistically significant differences (*p < 0.005; **p < 0.01).

Some pathogenic factors are responsible for regulating resistance to adverse environmental factors that fungal pathogens may encounter during their life cycle. Sensitivity to oxidative stress is associated with metabolic requirements such as purine biosynthesis and glutamine synthesis (Fernandez et al., 2013; Aron et al., 2021a). To investigate the contributions of the *MoAST2* gene in oxidative stress tolerance, we performed the fungal hyphal growth under H_2_O_2_ stress on CM media supplemented with 2.5-mM and 5-mM concentrations of H_2_O_2_. As expected, the hyphal growth of Δ*Moast2* was severely affected, but not as much in the wild-type and complementary strains (Figures 6A,B). The Δ*Mast2* strains were hypersensitive to 5 mM H_2_O_2_ concentrations (Figures 6A,B), implying the involvement of *MoAST2* in the tolerance of oxidative stress in *M. oryzae*.

Also, amino acid metabolisms were reported to be relevant to cell wall integrity. In fact, cell wall defects had been proved to be associated with the *MoGLN2* deletion in the rice blast fungus (Aron et al., 2021a). We analyzed the hyphal growth of Δ*Moast2* on CM medium supplemented with cell wall stressors Congo Red (CR) and Sodium Dodecyl Sulfate (SDS). However, similar to the Δ*Moast2/MoAST2* and wild-type strains, the Δ*Moast2* strains were jaded to cell wall stressors (Figures 6C,D), revealing the complicated relationship between amino acid metabolism and biological function.

### Δ*MoAST2* Is Auxotrophic to Glutamate Family, but Not Aspartate Family

**FIGURE 7:**
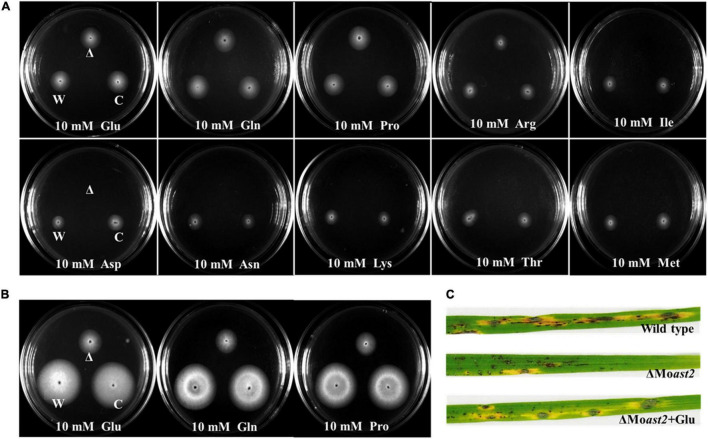
Auxotrophic phenotype of Δ*Moast2* on MM supplemented with individual amino acids. **(A)** The Δ*Moast2* mutant, wild-type, and complemented strains were inoculated on MM or MM plates supplemented with individual amino acids; 7 days after inoculation at 25°C. **(B)** H_2_O_2_ tolerance recovery of Δ*Moast2* on CM supplemented with selected amino acids 7 days after inoculation at 25°C. “C” represents complementary strains, “Δ” represents deletion mutant, “W” represents wild type. **(C)** Δ*Moast2* pathogenicity was recovered when conidial suspensions supplemented with glutamate were sprayed on rice leaves.

Generally, the double directions of the reversible reaction of glutamate/aspartate are all catalyzed by AST. To evaluate MoAST2 function involved in tolerance to nitrogen starvation, Δ*Moast2*, Δ*Moast2/MoAST2*, and the wild type were inoculated onto MM or MM supplemented with the indicated single amino acid as sole nitrogen source. The Δ*Moast2* mutant strains, compared to the Δ*Moast2/MoAST2* or wild-type strains, were almost unable to grow at Day 7 when applied with 10 mM aspartate or asparagine as the sole nitrogen source (Figure 7A), reflecting metabolism of aspartate or asparagine was blocked. However, when glutamate or glutamine was supplemented to MM, the defect in growth of Δ*Moast2* was restored to the level of the wild-type or Δ*Moast2/MoAST2* strains (Figure 7A). Meanwhile, we checked other members of aspartate family and glutamate family. As a result, the Δ*Moast2* strains could grow in the supplemented MM with individual member of glutamate family, but not grow in aspartate family MM. According to the aspartate metabolic pathway, we proposed that the direction of the AST-catalyzed reaction is from aspartate to glutamine.

To address whether exogenous amino acids could restore the Δ*Moast2* in H_2_O_2_ tolerance, we inoculated the three strains onto the CM, supplemented, respectively, with 5mM glutamate, glutamine, and proline under treatment of 5mM H_2_O_2_. The result indicated that exogenous amino acids of glutamate family could partially restore the growth of Δ*Moast2* (Figure 7B). Accordingly, rice seedling disease assays were performed by spraying the Δ*Moast2* spore suspension mixed with 5 mM glutamate. The result demonstrated that the replenishment of glutamate partially restored the ability to cause blast disease (Figure 7C).

### Similar to *MoAST2*, *CgAST2* Is Involved in Necrotrophic Development of *Colletotrichum graminicola*

The developmental process of *C. graminicola* at least involves the vegetative mycelium growth and the conidial and infection stages in one disease cycle. Compared to the mycelial stage and infection stage, *CgAST2* exhibited much higher transcriptional activities in the conidial stages. In addition, the expression level of *CgAST2* gene was up-regulated within 72 h after infection (Figure 2E). The similar expression patterns of *CgAST2* and *MoAST2* at different development stages implied their similar roles in development and pathogenesis.

**FIGURE 8:**
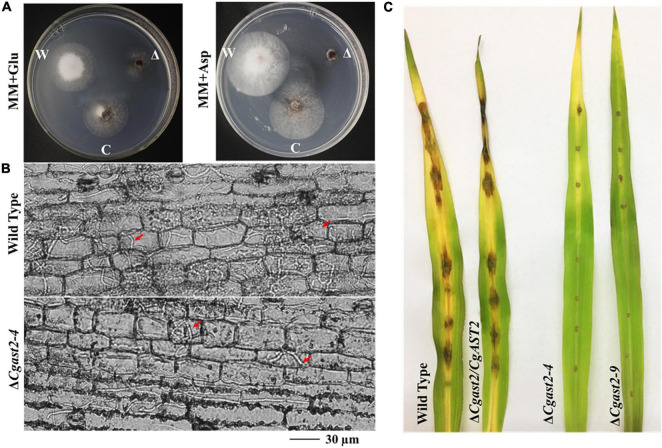
CgAST2, homolog of MoAST2, has a similar role in glutamate metabolism and pathogenesis. **(A)** The Δ*Cgast2* mutant could grow on MM-supplemented glutamate but not on MM with aspartate, 7 days after inoculation at 25°C. “C” represents complementary strains, “Δ”represents deletion mutant, “W” represents wild type. **(B)** Maize leaf infection assay. The conidial suspension of indicated strains was dropped onto a maize leaf. Representative photographs of infectious hyphae growing in the maize cell were taken after 72 h of incubation at 25°C. A bar indicates 30 μm. Red arrow points invasive hyphae. **(C)** Disease symptoms after the inoculation of leaves by spraying with conidia (1 × 10^5^/ml). Diseased leaves were photographed on 7 days post-inoculation.

Based on the significance of glutamate biosynthesis to virulence of *M. oryzae*, another hemibiotrophic fungal pathogen should be selected to check whether the *AST2* gene is conserved in pathogenesis. We therefore analyzed the maize anthracnose pathogen *C. graminicola* and identified *CgAST2*. We focused on investigating the biological functions of *CgAST2*. The Δ*Cgast2* mutant strains were generated (Supplementary Figure 3A). The Δ*Cgast2* strains were unable to grow on MM supplemented with aspartate (Figure 8A). When the Δ*Cgast2* strains were cultured on MM supplemented with 10 mM glutamate, like the Δ*Moast2*, the growth defects of Δ*Cgast2* could be reversed (Figure 8A). The result indicated that CgAST2 is required for glutamate biosynthesis.

In order to further examine the role of *CgAST2* in the infection process, we carried out microscopic observation after inoculating leaves. Like Δ*Moast2*, Δ*Cgast2*, together with Δ*Cgast2/CgAST2* and the wild type M1.001, all could form *in planta* invasive hyphae in the first infected cell. However, the Δ*Cgast2* strains, different from the wild type, were limited in the primary infected maize cells (Figure 8B). Even at 144hpi, Δ*Cgast2* could not result in typical symptoms on maize leaves (Figure 8C), suggesting *AST2* in pathogenesis is conserved.

## Discussion

Fungi are heterotrophic organisms. To most fungal saprophytes, nutrients including carbon and nitrogen, required for fungal growth and development, must be assimilated from around environments such as soil, dead plant or animal body, and organic residues. In general, the nature of available nutrition determines whether they can be absorbed and how much they can be absorbed. Take an example, ammonium is just preferred over nitrate (Marzluf, 1997; Wilson and Arst, 1998; Fernandez et al., 2012), and to some fungal pathogens, utilization of amino acids seems to be preferred over inorganic nitrogen uptake (Walters and Bingham, 2007). To the rice blast fungus *M. grisea*, the accelerated acquisition and utilization of nutrient were required for rapid proliferation of invasive hyphae at earlier infection stage (Parker et al., 2009). However, it appears that specific acquisition and utilization of certain amino acids are required during necrotrophic stage; due to that in *Colletotrichum lindemuthianum*, the nitrogen regulator (Clnr1) functioned specifically in necrotrophic pathogenesis but not in biotrophic pathogenesis (Pellier et al., 2003). Acquisition and utilization of amino acids are thus complex in different fungal pathogens.

On the other hand, to the fungal pathogens, more challenges are faced up in acquiring nutrients from a living host than in from dead organic matter. Pathogens must encounter nitrogen-starved threats during host plant-fungal pathogen interactions, particularly at biotrophic phase (Pellier et al., 2003; Wilson and Talbot, 2009). Thus, biosynthesis of amino acids is urgent for pathogenic fungi (Tang et al., 2019; Que et al., 2020). Biosynthesis of aspartate family of amino acids is required for pathogenesis in the rice blast fungus *M. oryzae*; due to that the gene deletion of synthesis enzymes or related regulation factors, required for most aspartate family members, resulted in the loss or reduction of pathogenicity (Divon and Fluhr, 2007; Rawal et al., 2014; Torres et al., 2016). At the necrotrophic phase, particularly to the hemibiotrophic pathogens such as *M. oryzae* or *C. graminicola*, what kind of amino acid is more important for the pathogenic *in planta* living? What metabolic pathways are beneficial to necrotrophic pathogenesis? In order to analyze the role of amino acids in necrotrophic pathogenesis, these questions need to be answered.

In the aspartate metabolic pathway, aspartate is the initial substrate that is very important in the biosynthesis of amino acids in prokaryotes, fungi, and some higher plants. It forms an early branch point in the metabolic pathway forming lysine, methionine, threonine, and isoleucine from aspartate (Van Bochaute et al., 2013). Our lab has focused on this pathway several years ago, and we have created a series of mutants including the disrupted mutant of the aspartate-semialdehyde dehydrogenase gene (*ASADH*), the downstream synthesis enzyme gene in *M. oryzae* (Liu, 2015). Indeed, loss of *ASADH* resulted in the impaired pathogenesis, which is just as the mutants of the other downstream synthesis enzyme genes reported in previous literature (Faehnle et al., 2006). Considering the reversible transfer of an α-amino group between aspartate and glutamate, catalyzed by aspartate aminotransferase (Figure 1), in this study, we provided genetic and biological evidence that the *MoAST2* was indeed essential for fungal growth, conidiation, and blast disease development. On minimal media, Δ*Moast2* was auxotrophic (Figure 7). In addition, aspartate, asparagine, and other aspartate family members did not restore the auxotrophic phenotype; however, when glutamate or glutamine was complemented in MM, Δ*Moast2* was capable of growing (Figure 7), demonstrating the aminotransferase activity from aspartate to glutamate. The leaf sheath inoculation assay indicated that the necrotrophic pathogenesis was impaired in Δ*Moast2* (Figure 5A) and Δ*Cgast2* (Figure 8C), suggesting that *AST2*-mediated glutamine biosynthesis, involved in necrotrophic pathogenesis, is conserved in plant fungal pathogens.

**FIGURE 9:**
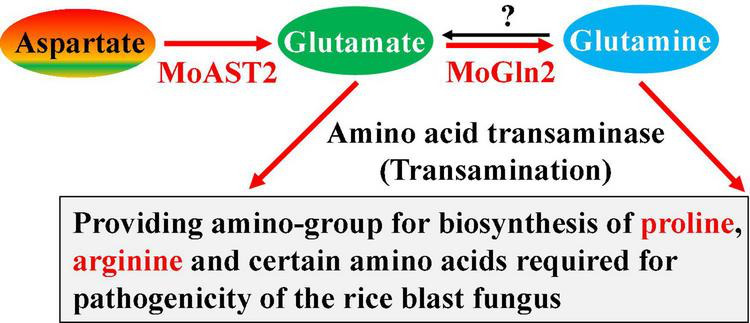
MoAST2-mediated pathogenic pathway. MoAST2 catalyzes aspartate transaminase to yield glutamate that then generate glutamine catalyzed by MoGln2, which as substrate enters biosynthesis of other glutamate family members. In this pathway, biosynthesis of glutamate, proline, and arginine have been confirmed to be important for pathogenesis in previous literatures.

Starting from the aspartate, there are three majority aspartate metabolic pathways (Figure 1), which appear to be involved in blast disease development, respectively. One is the very complicated pathway from aspartate to four terminal products: lysine, methionine, threonine, and isoleucine, which are, respectively, catalyzed by different enzymes (Du et al., 2013, 2014; Chen et al., 2014; Saint-Macary et al., 2015); another one is the simple one-step pathway from aspartate to asparagine, which is catalyzed by MoASN1; and the third pathway is from aspartate to glutamate, which was just the reported MoAST2 in this study (Figure 9).

Glutamate and glutamine serve as the major nitrogen source for other amino acid biosynthesis in most organisms. The glutamate family includes glutamate and other derived glutamine, proline, and arginine from glutamate; and lysine sometimes is included in this family. The biosynthesis of glutamate and glutamine is a key step in the nitrogen assimilation (Kwan et al., 2015; Dietl et al., 2020). Generally, the enzymes glutamine oxoglutarate aminotransferase and glutamate dehydrogenase catalyze the nitrogen assimilation reactions. However, in this research, the enzyme MoAST2 is able to transfer ammonia of aspartate to yield glutamate, replenishing glutamate. Proline and arginine are all derived from a glutamate through several steps of bioreactions driven by different key enzymes; therefore, glutamate biosynthesis determines the biosynthesis of proline and arginine. Three genes (MoARG1, MoARG5,6, and MoARG7) were involved in arginine biosynthesis of *M. oryzae*. Importantly, MoARG1, MoARG5,6, andMoARG7 are essential for growth, conidiogenesis, sexual reproduction, and pathogenicity in *M. oryzae* (Zhang et al., 2015). Recently, the carbamoyl phosphate synthase arginine-specific small chain subunit MoCpa1, which is required for arginine biosynthesis, was confirmed to be also crucial for fungal development, conidiation, appressorium formation, and infection-related morphogenesis in *M. oryzae* (Aron et al., 2021b). At this point, MoAST2-mediated pathogenesis should involve at least the contribution of arginine biosynthesis.

In terms of the aspartate-mediated pathogenic pathway, the three pathways appeared to be independent but necessary for pathogenesis, respectively, because the destruction of any single pathway would lead to serious pathogenic loss. Similar to the MoASN1-catalyzed reaction, MoAST2-catalyzed reaction is also a one-step reaction, but the MoAST2 is a reversible enzyme, different from the one-direction reaction of MoASN1 (the reverse-direction reaction needs asparaginase ASP1) (Marroquin-Guzman et al., 2018). However, MoAST2, in this report, did not possess the reverse-direction reaction activity. What factor controls the one-direction activity? Further study should focus on this question. Considering the recently reported MoGln2 (Aron et al., 2021a), which catalyzes glutamate to glutamine, we propose a novel pathogenic pathway mediated by MoAST2 and MoGln2 (Figure 9).

## Materials and Methods

### Fungal Strains and Growth Conditions

The wild-type strain *M. oryzae* JL0910 was previously isolated and purified from the rice cultivar Jijing88, which is widely planted in Jilin Province, China. All strains, including the four strains generated in this study, were cultured on CM agar plates and stored on filter paper at −20°C.

The strains were cultured for 7 days on CM for assessment of their growth rates. Each test was repeated at least three times. Liquid CM was used to prepare the mycelia for DNA and RNA. The mycelia used for nucleic acid or protein extraction were prepared by growing the relevant strains in 100 ml liquid CM for 3 days at 25°C with gentle rocking at 150 rpm under bright light. For conidiation, the strains were inoculated on oatmeal-tomato agar medium (OTA) and incubated at 25°C for 7 days in the dark (Saint-Macary et al., 2015). After the aerial hyphae of the colonies had been washed away using sterilized distilled water, the strains were continually grown for 3 days under a fluorescent light.

The *C. graminicola* M1.001 strain used as wild type (WT) in this study was a gift from Professor H.B. Deising (Institute of Agricultural and Nutritional Sciences, Martin Luther University Halle-Wittenberg, Halle, Germany). The wild-type, mutant, and complementation strains were cultured at 25°C. PDA was used for the growth and conidia production of the strains. Phenotypic tests were performed on complete minimal media. All the fungal strains used were maintained on paper filters at −20°C. *Agrobacterium tumefaciens* AGL-1 and *Escherichia coli* strains DH5α were cultured in lysogeny broth media. The pXEH2.0 vector to construct a recombinant vector, which was transformed into *A. tumefaciens* (AGL-1) competent cells. The AGL-1 strain was transformed with wild-type M1.001 using an *A. tumefaciens*-mediated transformation (ATMT) protocol (Münch et al., 2011).

### DNA and RNA Manipulations

Total RNA or DNA was extracted using RNA or DNA extraction kits (Sangon, Shanghai, China). The rice or maize leaves inoculated with *M. oryzae* or *C. graminicola* were collected at 24, 48, 72, 96, 120, and 144 hpi as samples (100 g) for total RNA extraction. First-strand cDNA was synthesized from 2.0 μg of total RNA using Avian Myeloblastosis Virus reverse transcriptase (Promega, Madison, WI, United States). The cDNA samples were diluted 10-fold and used as templates for PCR.

Quantitative real-time RT-PCR was run on an ABI 7500 Real-Time PCR System (Applied Biosystems, Foster City, CA, United States) following the manufacturer’s instructions. Reactions were performed in a 20-μl volume system. Transcriptions of genes were analyzed and the *ACTIN* gene was used as an endogenous control. Fold changes were calculated as 2^–ΔΔCt^ to analyze the relative abundance of transcripts. Quantitative real-time RT-PCR was repeated in triplicate with three independent biological experiments, and the primer pairs used in this section are listed in Supplementary Table 1.

### Generation Deletion of *MoAST2* or *CgAST2* Gene and Complementation Strains

To generate the *MoAST2* or *CgAST2* deletion strain Δ*Moast2* or Δ*Cgast2*, the *MoAST2* or *CgAST2* gene was replaced by the hygromycin resistant cassette (*HPH*). To construct the replacement vector, the flanking sequences were amplified with their corresponding primer pairs (MoAST-LB F/R and MoAST-RB-F/R) and fused with the HPH cassette in a pXEH knockout vector. The construct was confirmed using RT-PCR with the upstream forward and downstream reverse primers MoAST-F/R and MoAST-QF/R, and the confirmed construct was introduced into wild-type protoplasts. The *MoACTIN* gene (MGG_03982.6) was amplified with the primers MGG-Actin-Q-F/R to serve as an endogenous reference. The complementation fragments, which contain the entire *MoAST2* genes were amplified using PCR and inserted into the pKD7-RED (G418-resistance) vector or pCAMBIA1303 vector to complement Δ*Moast2* (Supplementary Figure 1).

To construct the DsRED-tagged *MoAST2* vector, *MoAST2* gene, a DNA fragment containing the full-length *MoAST2* open reading frame sequence was amplified with primers MoAST-PKD7-F/R. pKD7-RED contains the *DsRED* gene as a subcellular localization tag and the *G418* resistance gene as a selection marker. To construct the RFP-tagged *MoAST2* vector, *MoAST2* gene, a DNA fragment containing the full-length *MoAST2* open reading frame sequence was amplified with primers MoAST1303-F/R. pCAMBIA1303-GFP contains the *Green* fluorescent protein tag genes as a subcellular localization tag and the *G418* resistance gene as a selection marker (Supplementary Figure 1).

The same construction strategy was used to generate *CgAST2* deletion strains (Δ*Cgast2*). The primer pairs CgAST-LB-F/R and CgAST-RB-F/R were used to amplify the both flanking sequences, and the right DNA fragments were, respectively, fused with the HPH cassette in a pXEH knockout vector, obtained the deletion vector. For construction of the complementary vector of the Δ*Cgast2* strains, the pKD7-RED (G418-resistance) vector was used (Supplementary Figure 3). The corresponding PCR amplification primer pairs are CgAST-PKD7-F and CgAST-PKD7-R. All the primer sequences are listed in Supplementary Table 1.

### Conidiation Quantification, Appressorium Induction, and Rice Infection

After 10 days of cultivation on OTA, conidia were collected with 5 ml of distilled water, filtered through three layers of lens paper, and counted with a hemacytometer under a microscope. Conidial germination and appressorium formation were measured on a hydrophobic surface (plastic cover slips or Gel-bond films). Conidia suspensions of 30 μl (1 × 10^5^ spores/ml) were dropped onto a hydrophobic surface and were placed in a moistened box at 25°C. Appressorium formation rate was then calculated under the microscope at 12 hpi. More than 100 appressoria were counted for each strain and the experiment was repeated three times. Photographs were taken at 24 hpi.

### Fluorescence Microscopy

In order to observe the subcellular localization of the MoAST protein, *MoAST2-DsRED* or *MoAST2-GFP* was cultured for 7–14 days in the dark in a Potato Dextrose Agar (PDA) plate into which coverslips were obliquely inserted. The samples were observed using fluorescence microscopy until the mycelium extended to the coverslips.

### Stress Treatments and Cell Wall Integrity Test

To detect the effect of MoAST2 under exogenous H_2_O_2_, the wild-type, mutant, and complementation strains were continuously cultured on CM plates with concentrations of 0, 2.5, and 5.0 mM H_2_O_2_ in the dark for 7 days at 25°C, and the fungal colonies were observed and measured. Five-millimeter mycelial plugs were inoculated on CM plates with Congo red (CR) of 100 or 200 μg/mL, and SDS of 0.005% or 0.01% (w/v), respectively, and cultured in the dark at 25°C for 7 days to test the integrity of the cell wall. The growth of the colonies was observed, and the diameters were measured. These experiments were performed in triplicate and repeated three times for each strain.

### Amino Acid Assays

To determine the effect of selected amino acid auxotrophy on mycelium growth and pathogenicity, media and conidial suspension liquid were supplemented with 10 or 5 mM individual amino acid, at 28°C. Experiments were performed in triplicate.

### Plant Infection Assays

The rice cultivar Jijing88 was used for infection assays in identifying pathogenesis of *M. oryzae*. For the inoculation of the intact rice leaves, a conidial suspension (1 × 10^5^ conidia/ml) was sprayed onto the leaves using an air sprayer. The inoculated plants were placed in a high humidity chamber at 25°C for 24 h in the dark then transferred to a growth chamber with a 16-h light/8-h dark photoperiod. For microscopic observation of cuticle penetration and infectious hyphae growth, leaf sheaths and inoculation were prepared and inoculated with 100 ml of conidial suspension (1 × 10^5^ conidia/mL) on the inner leaf sheath cuticle cells. After 24, 48, and 72 h and incubation under humid conditions at room temperature, the leaf sheaths were observed under a microscope.

The corn cultivar used in the pathogenicity experiment was “Xianyu 335,” which is a variety that is planted widely in north China. The leaf spot inoculation test utilized 10 μl 5 × 10^5^ conidia/mL (2% gelatin, w/v) droplets from the wild-type, mutant, and complementation strains that were placed separately on the surfaces of third corn leaves. After inoculation, the leaves were placed in an artificial climate box with a relative humidity of 90% at 25°C and incubated in the dark for the first 24 h and then cultivated continuously for 6 days in the dark followed by light for 12 h each before examination. The leaves after spot inoculation were sampled at 24, 48, and 72 h, respectively, to microscopically observe the infection process. The samples were then sliced and observed with an optical microscope for pathogenicity.

### Statistical Analysis

All experiments were repeated at least three times. The mean ± standard deviation of the colony diameter, germination rate, and relative expression were determined using the Prism version 7.00 software (GraphPad, San Diego, CA, United States). The data were analyzed using InStat3 (GraphPad). The threshold for statistical significance was *p* < 0.05.

## Data Availability Statement

The original contributions presented in the study are included in the article/Supplementary Material, further inquiries can be directed to the corresponding authors.

## Author Contributions

S-HZ: conceptualization, writing—review and editing the manuscript, supervision, and funding acquisition for the research. PZ, ZF, and YW: editing original draft preparation, methodology, conceptualization, and data curation. YD, YS, and LB: data curation, investigation, validation, and project administration. SW and ML: formal analysis and software. All authors have read and agreed to the published version of the manuscript.

## Conflict of Interest

The authors declare that the research was conducted in the absence of any commercial or financial relationships that could be construed as a potential conflict of interest.

## Publisher’s Note

All claims expressed in this article are solely those of the authors and do not necessarily represent those of their affiliated organizations, or those of the publisher, the editors and the reviewers. Any product that may be evaluated in this article, or claim that may be made by its manufacturer, is not guaranteed or endorsed by the publisher.

## Footnotes

1
http://fungi.ensembl.org/Magnaporthe_oryzae/Tools/blast

2
https://www.ncbi.nlm.nih.gov/

3
http://zhanglab.ccmb.med.umich.edu/I-TASSER/

4
http://www.csbio.sjtu.edu.cn/cgi-bin/EukmPLoc2.cgi
