# Supplementary material for: Aspartate Transaminase AST2 Involved in Sporulation and Necrotrophic Pathogenesis in the Hemibiotrophs Magnaporthe oryzae and Colletotrichum graminicola
Source: Front Microbiol. 2022 Apr 11;13:864866. doi: 10.3389/fmicb.2022.864866 (PMC9037547; doi:10.3389/fmicb.2022.864866)
Supplement: Supplementary file 1 [file Data_Sheet_1.docx]

**Table S1. Primers used for PCR amplification in this research**

| **Primer name** | **Sequence (5΄→3΄) (restriction site at 5-end)** | **Use** |
| --- | --- | --- |
| MoAST-LB-F | GAAGATCTGCTTTTCTAGTAATCTGGGACC | *MoAST2* deletion |
| MoAST-LB-R | GGACTAGTAAAGGGGTGTTGAGTAGGTAAA | *MoAST2* deletion |
| MoAST-RB-F | CGGGATCCAGTAGCTGCAGATACTACTATGTCG | *MoAST2* deletion |
| MoAST-RB-R | CCAAGCTTAGCCGTCCAGAAGTCTTATTTTA | *MoAST2* deletion |
| MoAST-F | ATGTCGGCAAAAGCCACCC | *MoAST2* cloning |
| MoAST-R | TTATTGCGTCTCCCTCACCACCTTA | *MoAST2* cloning |
| HYG-F | GATGTAGGAGGGCGTGGATATGTCCT | Hygromycin detection |
| HYG-R | AACCCGCGGTCGGCATCTACTCTATTC | Hygromycin detection |
| MoAST-QF | CCATACTTTGACAAGGGCACC | *MoAST2* identification |
| MoAST-QR | TAAGCACAGTCGAAAAACGGG | *MoAST2* identification |
| MGG-Actin-QF | CCATGTACCCTGGTCTTTCG | Gene expression normalization |
| MGG-Actin-QR | TTCGAGATCCACATCTGCTG | Gene expression normalization |
| MoAST-PKD7-F | GCTCTAGAATGTCGGCAAAAGCCACCC | Subcellular location and complementation |
| MoAST-PKD7-R | GCTCTAGATTATTGCGTCTCCCTCACCACCTTA | Subcellular location and complementation |
| MoAST1303-F | CATGCCATGGATGTCGGCAAAAGCCACCC | Subcellular location and complementation |
| MoAST1303-R | GACTAGTTTATTGCGTCTCCCTCACCACCTTA | Subcellular location and complementation |
| MoAST-HYG-LB-F | GAGGAGAAGAAGCGAGGACC | Transformant confirmation |
| MoAST-HYG-LB-R | TCATTTGGATGCTTGGGTAGA | Transformant confirmation |
| MoAST-HYG-RB-F | TGGTTCATTTAGGCAACTGGTC | Transformant confirmation |
| MoAST-HYG-RB-R | TGGCGTTGTGAGAAGGTTTG | Transformant confirmation |
| CgAST-LB-F | GAAGATCTGATGGATAACCTGGTGAAC | *CgAST2* deletion |
| CgAST-LB-R | GACTAGTGAGCTGGAAGCTGCTAAG | *CgAST2* deletion |
| CgAST-RB-F | CGGGATCCGGTGTGTTATTATGTGTTTGTA | *CgAST2* deletion |
| CgAST-RB-R | ACGCGTCGACGTTGATAGTGACCCGTCGCG | *CgAST2* deletion |
| CgAST-LB-HYG-F | TACCATACTTACTTTCTCCCTG | Transformant detection |
| CgAST-LB-HYG-R | GATGATAATAATGTCCTCGTTC | Transformant detection |
| CgAST-LB-HYG-F | ACTGATATTGAAGGAGCA | Transformant detection |
| CgAST-LB-HYG-R | TATGGACAAAGCACTAGA | Transformant detection |
| CgAST-F | ATGCCCGGTGACTCCTCCT | *CgAST2* cloning |
| CgAST-R | CTGTGTCTCGCGGACGACC | *CgAST2* cloning |
| CgAST-PKD7-F | GCTCTAGAATGCCCGGTGACTCCTCCT | *CgAST2* complementation |
| CgAST-PKD7-R | AACTGCAGCTGTGTCTCGCGGACGACC | *CgAST2* complementation |
| CgAST-Q-S | TACGCAGTATCGCATCAC | Gene expression detection |
| CgAST-Q-R | CTGTAGGCAGCCATCAAG | Gene expression detection |
| Cg-actin-F | GATTTGGCACCACACTTTC | Gene expression normalization |
| Cg-actin-R | TCTTCTCTCTGTTGGACTT | Gene expression normalization |

Figure S1. **The construction strategies for** *MoAST2* **deletion and complementation strains**

(A) Strategy for construction of the *MoAST2* deletion mutant Δ*Moast2*. The *MoAST2* gene was deleted by substituting the *MoAST2* DNA fragment with a 1.4-kb *HPH* cassette from the pXEH vector. The two flanking regions were ligated to both sides of the *HPH* cassette in the pXEH vector. The primer pair MoAST-HYG-LB-F/R and MoAST-HYG-RB-F was used to confirm the final construct. (B) Based on the pCAMBIA1303 vector, *MoAST2* amplified from genomic DNA, was inserted into the MCS, constructing the sublocalization vector. (C) DsRED-tagged MoAST2 was created through the similar strategy described above. Corresponding primer pairs were listed in Table S1.

**Figure S2. Vegetative growth phenotype analysis of Δ*MoAST2***

The Δ*Moast2*, Δ*Moast2*/*MoAST2*, and the wild type strains on CM. After 7 days of cultivation, mycelial growth was analyzed according to the colony diameter among the three strains. (A) Colony growth (view from the front of the plate) of tested strains. (B) Measurement of the colony diameter of tested strains. (C) Colony pigmentation (view from the back of the plate) of tested strains. (D) pigment alteration in liquid culture. Asterisks indicate statistically significant differences (*P < 0.05; Data represent the means ± standard deviation from three independent experiments in which triplicate plates were examined for each strain in each experiment).

**Figure S3. The construction strategies for *CgAST2* deletion and complementation strains**

(A) The *CgAST2* gene was replaced by the hygromycin resistant cassette (HPH). To construct the replacement vector, the flanking sequences were amplified with their corresponding primer pairs and fused with the *HPH* cassette. (B) DsRED-tagged CgAST2 vector. Details of the construction are described in Materials and Methods.

**Fig S1.**


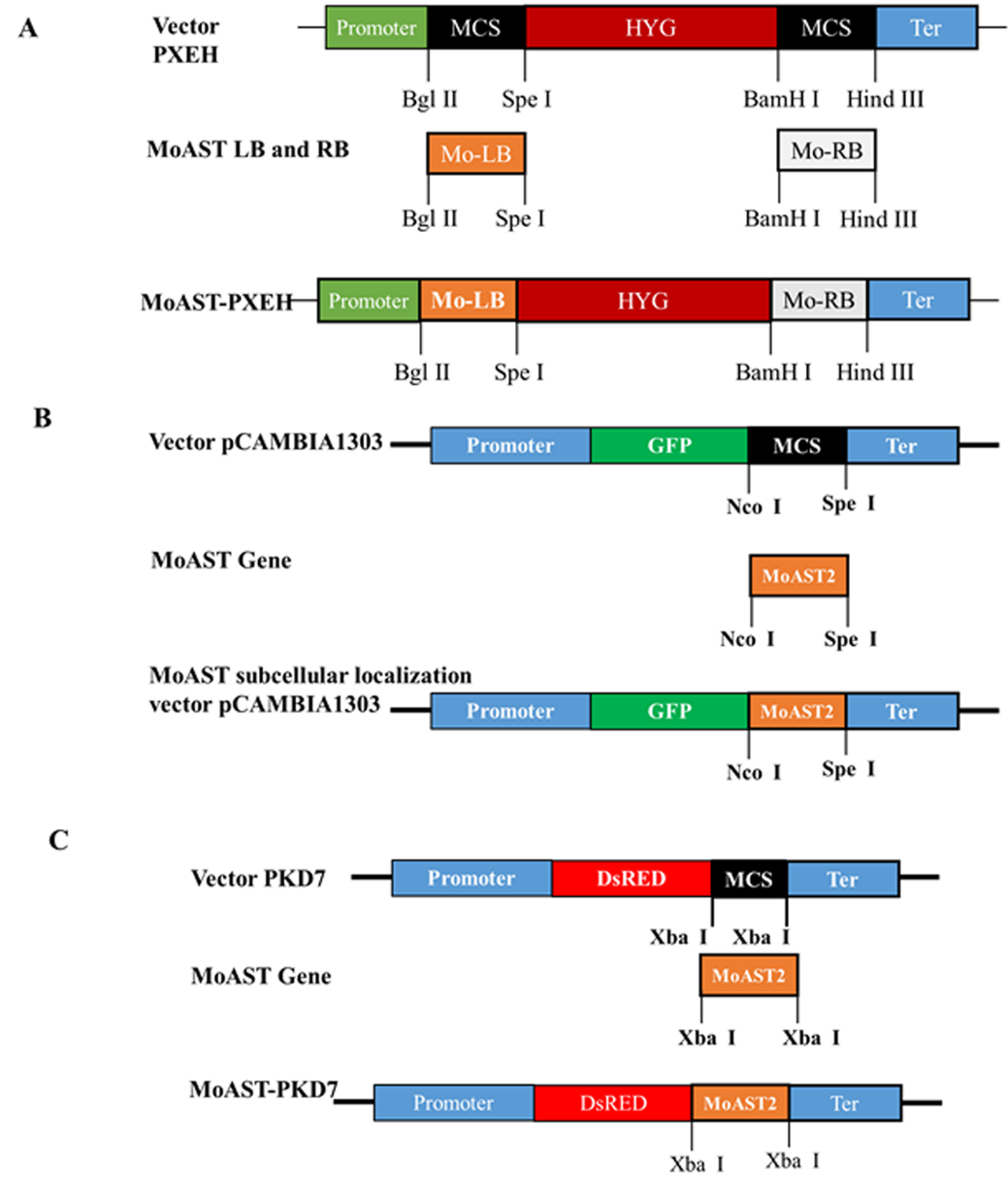


**Fig S2.**


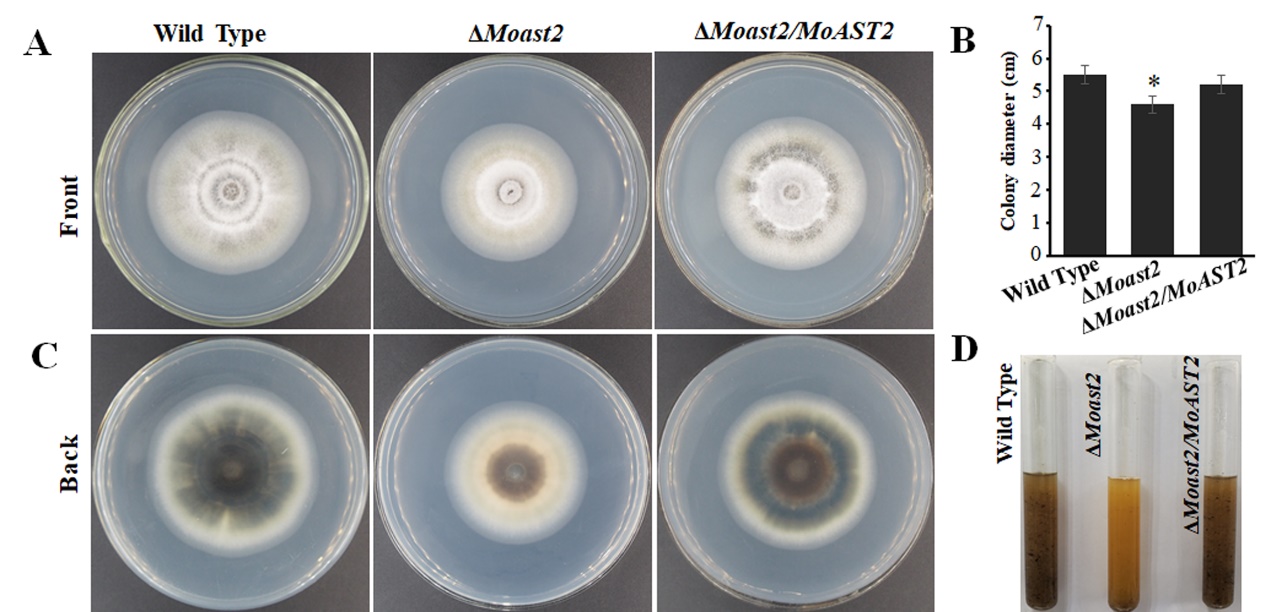


**Fig S3.**

**
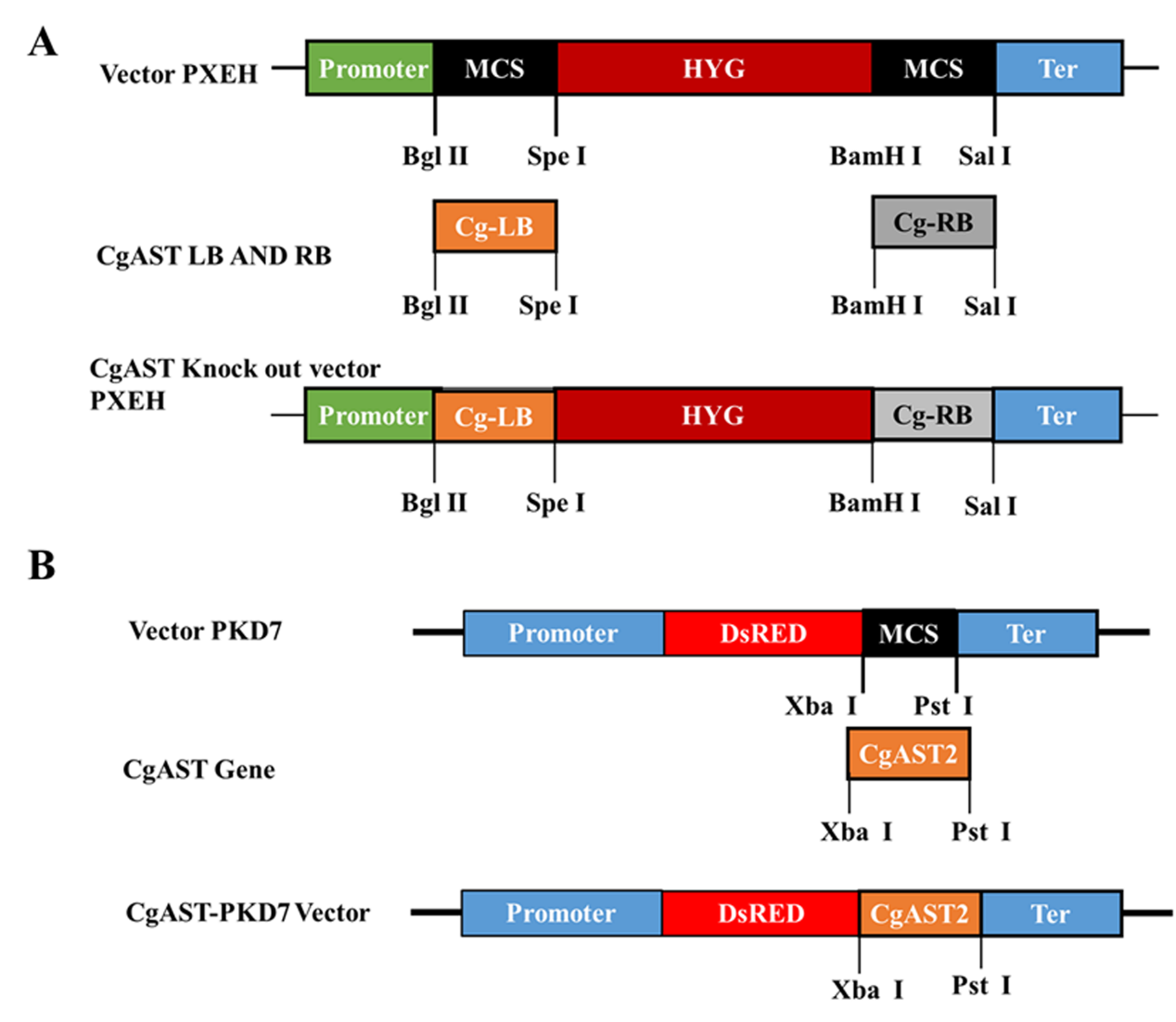
**
